# Rare Earth Elements Alter Redox Balance in *Methylomicrobium alcaliphilum* 20Z^R^

**DOI:** 10.3389/fmicb.2018.02735

**Published:** 2018-11-27

**Authors:** Ilya R. Akberdin, David A. Collins, Richard Hamilton, Dmitry Y. Oshchepkov, Anil K. Shukla, Carrie D. Nicora, Ernesto S. Nakayasu, Joshua N. Adkins, Marina G. Kalyuzhnaya

**Affiliations:** ^1^Biology Department, Viral Information Institute, San Diego State University, San Diego, CA, United States; ^2^Institute of Cytology and Genetics SB RAS, Novosibirsk, Russia; ^3^Novosibirsk State University, Novosibirsk, Russia; ^4^Biological Sciences Division, Pacific Northwest National Laboratory, Richland, WA, United States

**Keywords:** *Methylomicrobium alcaliphilum* 20Z^R^ strain, methanol dehydrogenase, MxaFI, XoxF, transcriptomics, proteomics, metabolomics

## Abstract

**Background:** Rare Earth Elements (REEs) control methanol utilization in both methane- and methanol-utilizing microbes. It has been established that the addition of REEs leads to the transcriptional repression of MxaFI-MeDH [a two-subunit methanol dehydrogenase (MeDH), calcium-dependent] and the activation of XoxF-MeDH (a one-subunit MeDH, lanthanum-dependent). Both enzymes are pyrroquinoline quinone-dependent alcohol dehydrogenases and show significant homology; however, they display different kinetic properties and substrate specificities. This study investigates the impact of the MxaFI to XoxF switch on the behavior of metabolic networks at a global scale.

**Results:** In this study we investigated the steady-state growth of *Methylomicrobium alcaliphilum* 20Z^R^ in media containing calcium (Ca) or lanthanum (La, a REE element). We found that cells supplemented with La show a higher growth rate compared to Ca-cultures; however, the efficiency of carbon conversion, estimated as biomass yield, is higher in cells grown with Ca. Three complementary global-omics approaches–RNA-seq transcriptomics, proteomics, and metabolomics–were applied to investigate the mechanisms of improved growth vs. carbon conversion. Cells grown with La showed the transcriptional activation of the *xoxF* gene, a homolog of the formaldehyde-activating enzyme (*fae2*), a putative transporter, genes for hemin-transport proteins, and nitrate reductase. In contrast, genes for *mxaFI* and associated cytochrome (*mxaG*) expression were downregulated. Proteomic profiling suggested additional adjustments of the metabolic network at the protein level, including carbon assimilation pathways, electron transport systems, and the tricarboxylic acid (TCA) cycle. Discord between gene expression and protein abundance changes points toward the possibility of post-transcriptional control of the related systems including key enzymes of the TCA cycle and a set of electron-transport carriers. Metabolomic data followed proteomics and showed the reduction of the ribulose-monophosphate (RuMP) pathway intermediates and the increase of the TCA cycle metabolites.

**Conclusion:** Cells exposed to REEs display higher rates of growth but have lower carbon conversion efficiency compared to cells supplemented with Ca. The most plausible explanation for these physiological changes is an increased conversion of methanol into formate by XoxF-MeDH, which further stimulates methane oxidation but limits both the supply of reducing power and flux of formaldehyde into the RuMP pathway.

## Introduction

Methanotrophs are promising systems for mitigating greenhouse gas emissions, enhancing bioremediation, and producing feed, fuel, and chemicals (Kirschke et al., 2013; Strong et al., 2016; Handler and Shonnard, 2018). This growing interest in environmental or commercial applications has directed research toward a system-level understanding of biological methane utilization (Karlsen et al., 2011; Matsen et al., 2013; Yang et al., 2013; de la Torre et al., 2015; Akberdin et al., 2018).

The metabolic network of methane oxidation is surprisingly redundant. To consume methane, a methanotroph must have at least one of the two monooxygenases (MMO) for methane oxidation: a particulate or membrane bound form of MMO (pMMO) and/or a soluble MMO (sMMO) which is compartmentalized into cytoplasm. Both enzymes require oxygen and an additional source of reducing power for methane activation, and both convert methane to methanol and water. The second metabolic reaction is catalyzed by a pyrroquinoline quinone (PQQ)-linked methanol dehydrogenase (MeDH) (Myronova et al., 2006; de la Torre et al., 2015; Semrau et al., 2018). At least two forms of MeDH have been described: a calcium-containing two-subunit MeDH, MxaFI-MeDH, and, an alternative single-subunit enzyme, XoxF-MeDH (Fitriyanto et al., 2011; Hibi et al., 2011; Nakagawa et al., 2012; Pol et al., 2014; Haque et al., 2015). Several metabolic routes can contribute to formaldehyde oxidation (Chistoserdova, 2011), and up to four formate dehydrogenases can contribute to the final step of methane oxidation (Chistoserdova et al., 2004; Chistoserdova and Kalyuzhnaya, 2018).

Numerous microelements have been established or are newly emerging as control points for primary methane oxidation (Glass and Orphan, 2012; Chidambarampadmavathy et al., 2015; Semrau et al., 2018). Three key metabolic switches have been described: (1) a copper-switch, which controls the expression and activity of primary methane oxidation (Stanley et al., 1983; Semrau et al., 2018); (2) a tungsten-molybdenum (W/Mo) switch for formate oxidation (Laukel et al., 2003; Chistoserdova et al., 2004; Akberdin et al., 2018); and (3) a La-switch, which negatively regulates the expression of MxaFI-MeDH and activates XoxF-MeDH (Haque et al., 2015; Chu and Lidstrom, 2016; Chu et al., 2016; Gu and Semrau, 2017; Semrau et al., 2018). Initial evidence with microbial systems that have all three types of these metabolic switches highlights the complexity of metabolic responses and suggests crosstalk between copper and REE pathways (Gu and Semrau, 2017; Semrau et al., 2018). Furthermore, substitutions at the level of a single metabolic step are not always metabolically neutral and can impact the overall cellular network. For example, a lack of copper is linked to a change from pMMO to sMMO, which leads to a significant drop in carbon conversion efficiencies and growth rates (Leak and Dalton, 1986; DiSpirito et al., 2016; Kenney and Rosenzweig, 2018). This change could be linked to the specific requirement of sMMO for NADH, which contributes to the redox limitation upon copper starvation (Leak and Dalton, 1986). Differences in growth rate and/or biomass yield have also been noted for a switch from Ca to REEs for some methylotrophic bacteria (Vu et al., 2016; Good et al., 2018; Masuda et al., 2018). It has been demonstrated that the expression of MxaFI-MeDH only occurs in the absence of La, making XoxF-MeDH a more preferable system for carbon utilization in microbes using the serine cycle pathway for carbon utilization (Good et al., 2018). However, it still remains unclear why the substitution of one PQQ-dependent dehydrogenase with another functionally similar PQQ-dependent dehydrogenase impacts overall carbon utilization. Both enzymes can convert methanol to formaldehyde and formaldehyde to formate *in vitro* (Anthony and Williams, 2003; Schmidt et al., 2010; Keltjens et al., 2014; Huang et al., 2018), but whether this is true *in vivo* remains controversial. The activity of MxaFI-MeDH could be modulated, making formaldehyde the main product (97%) *in vivo* (Page and Anthony, 1986). The enzyme couples methanol oxidation with the reduction of cytochrome c_L_, which passes electrons to cythocrome c_H_, and then to a cytochrome oxidase (Anthony and Williams, 2003). The overall balance of the reaction could be presented as following:

CH3OH+12O2+0.5−1 ATP+0.5−1Pi=CH2O+H2O+0.5−1 ATP

Dual activity, methanol-to-formaldehyde and formaldehyde to-formate, has been proposed for the XoxF-MeDHs *in vivo* (Keltjens et al., 2014). If the dual activity indeed occurs, the overall balance could be summarized as:

$${\mathrm{CH}}_{3}\mathrm{OH}+O_{2}+1-2\;\mathrm{ATP}+1-2\;\mathrm{Pi}=\mathrm{CHOOH}+H_{2}O+1-2\;\mathrm{ATP}$$

While in verrucomicrobial methanotrophs (assimilating carbon via the Rubisco pathway) as well as alphaptoteobacterial methanotrphs (assimilating carbon from formate), the dual activity does not directly impact carbon assimilation, it could be predicted that in methanotrophs with the formaldehyde assimilation pathways the dual methanol/formaldehyde activity can lead to several metabolic challenges, including redox limitation and restriction of formaldehyde flux into C_1_-assimilation. The global metabolic consequences of a MxaFI-MeDH to XoxF-MeDH swap in microbes possessing both systems remain to be investigated. Nevertheless, XoxF-MeDH has been described as the preferred system for methane and methanol utilization (Chu et al., 2016; Yu et al., 2017; Huang et al., 2018). Five families of XoxF-MeDH homologs have been described, and it is becoming apparent that they display different catalytic properties and might be linked to different electron transport systems (Yu et al., 2017; Huang et al., 2018; Zheng et al., 2018). Some XoxF’s cluster together with cytochrome-like genes; the electron acceptors for others are not apparent. Among the latter are the XoxF5-MeDHs found in gammaproteobacterial methanotrophs. An association between XoxF5 and a cytochrome b1 homolog (*xoxG4*) has been proposed (Yu et al., 2017); however, expression of the cytochrome does not parallel *xoxF* expression in *Methylomonas* LW13 and an *xoxG*4-mutant shows a strikingly different phenotype (Huang et al., 2018), indicating that an alternative electron-transfer partner (or partners) must be coupled with XoxF5-MeDH (Huang et al., 2018).

In this study, we examine the metabolic response of *Methylomicrobium alcaliphilum* 20Z^R^ to REEs at the global scale via transcriptomic, proteomic and metabolomic studies. *M. alcaliphilum* 20Z^R^ has only one enzyme for methane oxidation (pMMO, copper dependent), two MeDHs (MxaFI-MeDH and XoxF5-MeDH), and only one tungsten-dependent formate dehydrogenase and thus it represents a good model for investigating the REE-mediated switch independently from copper or W/Mo responses.

## Results

### Ca vs. La: Growth Parameters

Two continuous cultures of *M. alcaliphilum* 20Z^R^ were set up as described in Material and Methods and the main growth parameters are summarized in Table 1 and Supplementary Figure S1. The steady-state growth of the Ca-supplemented culture was established as a specific growth rate of 0.05 h^-1^ was observed for both, 5% CH_4_ : 5%O_2_ (optimal) and 2.5% CH_4_ : 10%O_2_ (methane-limited) gas supply. The growth rate was higher for the La-supplemented culture, reaching 0.07 h^-1^ and 0.06 h^-1^ at optimal and methane-limited inputs, respectively. The overall biomass yield (Y_B_) reached 1.2 in Ca-supplemented cultures and 0.67 in cultures supplemented with La. Oxygen consumption also differed between Ca and La conditions, with cells grown with La consuming more oxygen per methane converted compared to cells grown with Ca (1.28 vs. 1.12). Reduction in the methane supply and/or an increased O_2_ supply ratio led to a 1.8-fold reduction in the growth rate of the La-supplemented cells (Table 1). Samples of cells grown at optimal conditions and methane-limiting conditions were used for gene expression studies. All other *omics-*studies were done only with samples of cells grown at optimal conditions.

**Table 1:** Growth parameters and substrate consumption in continuous bioreactor cultures of *M. alcaliphilum* 20Z^R^ supplemented with Ca or La.

Growth parameters	Ca		La	
		
	5%CH_4_ : 5% O_2_	2.5%CH_4_ : 10%O_2_	5%CH_4_ : 5% O_2_	2.5%CH_4_ : 10%O_2_
Dilution rate^∗^ (h^-1^)	0.05	0.05	0.07	0.06
Biomass^∗^ (g DCW L^-1^)	0.64 ± 0.01	0.67 ± 0.02	0.75 ± 0.05	0.45 ± 0.02
Biomass yield (g biomass g^-1^ CH_4_ consumed)	1.2 ± 0.1	0.98 ± 0.04	0.64 ± 0.01	0.67 ± 0.03
O_2_:CH_4_ consumption ratio	1.12 ± 0.09	NT	1.28 ± 0.01	NT
CH_4_ consumption (mmol g^-1^ DCW h^1^)	2.59 ± 0.26	3.11 ± 0.11	6.75 ± 0.09	5.55 ± 0.2
Biomass produced (mg DCW h^-1^)	31.8 ± 0.5	33.7 ± 1.02	53.1 ± 0.7	28.7 ± 1.1


*NT: not tested; ^∗^dilution rate or cell concentration at steady-state.*

### Ca vs. La: Gene Expression Profiles

Samples of bioreactor cultures (two biological replicates per tested growth condition) were collected for generating gene-expression profiles using RNA-sequencing technology. Transcriptomes of replicates for both growth conditions are highly similar; the Pearson’s correlation between the two replicates for both Ca-added and La-added samples was >0.98. Over 800 genes were found to have statistically significant differential expression between the two growth conditions (i.e., a Benjamini-Hochberg adjusted *p*-value < 0.05) with 150 genes having a | log2| change ≥1.5 (Table 2 and Supplementary Table S1).

**Table 2:** Heatmap comparing the differentially expressed genes between Ca -and La-cultures.

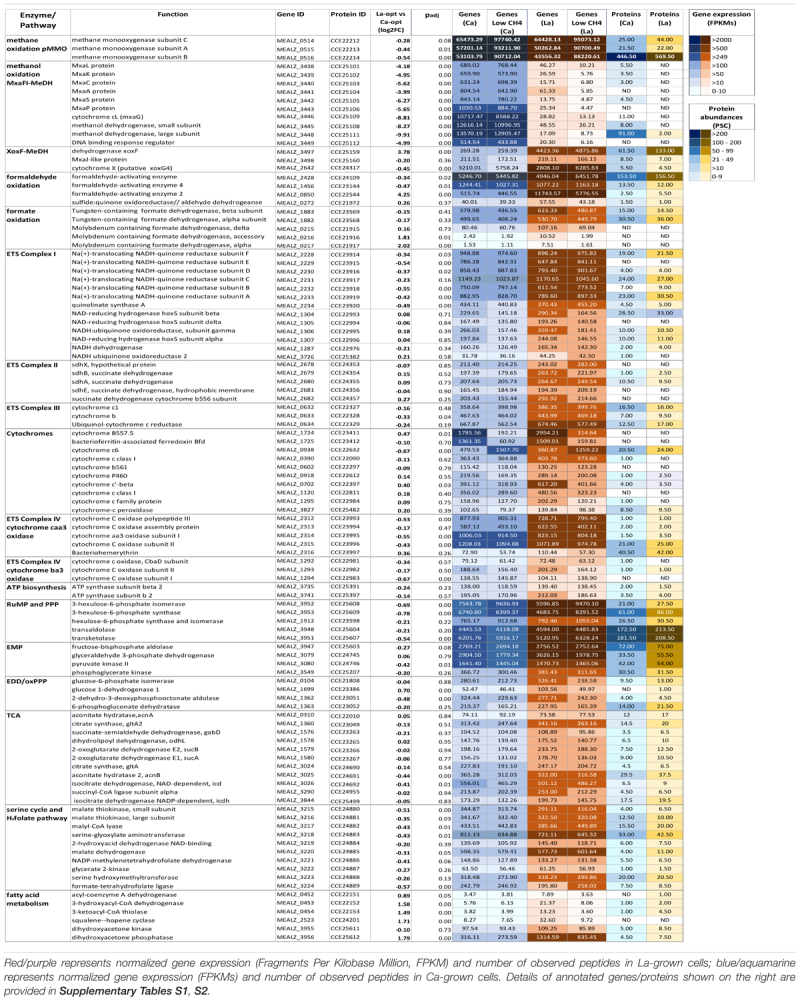

Twenty-four genes were identified as significantly downregulated when the 20Z^R^ culture was supplemented with La instead of Ca (Supplementary Table S1). The set includes 13 genes encoding the two-subunit MeDh MxaFi, its corresponding cytochrome, proteins essential for the enzyme’s assembly and folding, and its response regulator, MxaB (MEALZ_3449). Among the other downregulated genes were two genes (MEALZ_3990, MEALZ_3991) which encode the MotA/TolQ/ExbB proton channel family protein. CorA (MEALZ_2831) and corB (MEALZ_2832) genes, predicted to encode a copper-repressible surface-associated protein and associated di-haem cytochrome c peroxidase (Karlsen et al., 2010; Shchukin et al., 2011; Johnson et al., 2014) were also downregulated in the presence of La.

**FIGURE 1:**
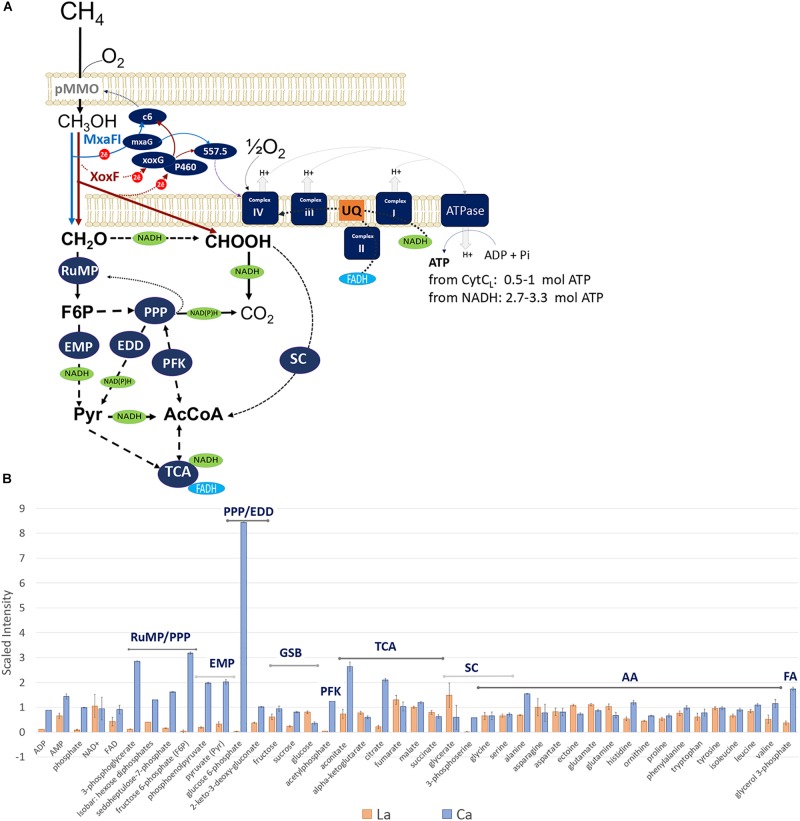
**(A)** Central metabolic pathways of *M. alcaliphilum* 20Z^R^. All abbreviations of metabolic pathways and enzymes are presented in the main text. **(B)** Compounds detected in cells grown with La (red) or Ca (blue).

A larger number of genes (126, representing 98 operons) were upregulated when La was added instead of Ca to the growth medium (Supplementary Table S1 and Figure 1). A significant portion of these genes are represented by hypothetical proteins. Among genes with predicted cellular functions are the alternative mono-subunit MeDH gene, *xoxF* (MEALZ_3497), whose expression increased by fourfold; a putative formaldehyde-activating enzyme (*fae2*) gene; an operon of genes encoding delta (*fds2D*), gamma (*fds2C)* and a partial alpha subunit (*fds2A)* of molybdenum-dependent formate dehydrogenase (Fds2); beta-oxidation pathways (FadAB) of fatty acids; and squalene-hopene cyclase. Among other annotated genes responding to the presence of La are two sets of genes homologous to urea ABC transporters and the sulfate transport system, respectively.

The expression of *xoxG*4 (MEALZ_2642), the putative cytochrome b proposed to accept electrons from the XoxF5 enzyme (Yu et al., 2017), was reduced 1.5-fold compared to Ca-grown cells (Supplementary Table S1). The expression of the cytochrome could be correlated with methane limitation rather than with La-growth (Figure 1). From 22 cytochromes identified in the genome of *M. alcaliphilum* 20Z^R^, four,—cytochrome P460 (MEALZ_0918), cytochrome c1-type (MEALZ_1120), cytochrome c’-beta (MEALZ_0702) and cytochrome B557.5 (MEALZ_1724) with associated ferredoxin (MEALZ_1724)—responded positively to the addition of La.

### Ca vs. La: Proteomics Data

Samples of cell cultures were also used to investigate protein profiles at the same growth time points used for transcriptomics analyses. More than twenty-seven hundred proteins were identified by quantitative proteomic analysis (Figure 1, Table 2, and Supplementary Table S2).

In general, the proteomic data correlated well with the gene-expression profiles for cells grown with La, showing lower levels of MxaFI-MeDH and associated cytochrome and accessory proteins than Ca-grown cells (Table 2 and Supplementary Table S2).

Also in agreement with transcriptomic profiles, XoxF5-MeDH, formaldehyde-activating enzyme 2 and 3-ketoacyl-CoA thiolase abundances increased in response to La. No change in XoxG4 abundance was observed.

However, several differences between transcriptomics and proteomics datasets were observed (Figure 1, Table 2, and Supplementary Tables S1, S2). Among them are enzymes/accessory proteins involved in the central pathways of C_1_-assimilation (methenyltetrahydromethanopterin cyclohydrolase), the TCA/serine cycle (malate dehydrogenase), amino acid metabolism (chorismate synthase, tryptophan synthase subunit beta) and electron transport systems (cytochrome c oxidase subunit I, cytochrome bc1 and cytochrome P460)—all showing protein-abundance increases with La without significant changes in gene expression.

### Ca vs. La: Metabolic Switches

Non-targeted metabolic profiling was then applied to further investigate the consequences of the switch to REEs on cellular metabolism (Supplementary Table S3). The intermediates of the central metabolic pathways including the RuMP pathway (sedoheptulose-7 phosphate, fructose-6 phosphate, glucose-6 phosphate, phosphoenolpyruvate, 3-phosphoglycerate) and the first two steps of the TCA cycle (aconitate, citrate) dropped down significantly in La-grown cells compared to Ca-grown cells (Figure 1B), while concentrations of the TCA/serine cycle intermediates (fumarate, malate, and succinate) did not significantly change or slightly increased. The intracellular pools of amino acids produced from the TCA intermediates (glutamate, glutamine, asparagine, and ectoine), the key serine cycle intermediate (glycerate) also increased (Figure 1B).

Highly elevated levels of agmatine in cells grown on La could be linked to the upregulation of the urea ABC transporter permease because the substance is a precursor for urea biosynthesis. However, no urea was detected in supernatant samples even with targeted metabolite detection methods (see Material and Methods).

### Ca vs. La: Flux Balance (FBA) Simulations

Cell growth performance and metabolite data suggest that MxaFI-MeDH to XoxF-MeDH changes behavior of all central metabolic pathways downstream from methanol oxidation, indicating that the enzymes somehow differ in their functions. One possible explanation is that XoxF-MeDH has a higher affinity for its product (formaldehyde) and can convert formaldehyde to formate (Schmidt et al., 2010). Hence, the impact of the two-step conversion was tested *in silico*. The La-switch in *M. alcaliphilum* 20Z^R^ could be associated with a number of changes in the main physiological outputs, including the acceleration of O_2_-consumption. The increase would indicate changes in redox balance and the acceleration of respiratory pathways. Taking into account that XoxF-MeDHs, including XoxF5, can convert both methanol and formaldehyde (Schmidt et al., 2010; Huang et al., 2018; Masuda et al., 2018) the La-switch could increase production of a reduced cytochrome instead of NADH (Figure 1B). To simulate the behavior of metabolic networks upon La-perturbation, we modified a previously developed computational model of methane metabolism (Akberdin et al., 2018) and incorporated a cytochrome-mediated formaldehyde oxidation reaction. *In silico* and observed O_2_/CH_4_ consumption ratios reached an agreement when 25% of formaldehyde pool is directed toward formate via a cytochrome-linked enzyme, such as XoxF-MeDH (Table 3).

**Table 3:** Flux balance simulations of methanotrophic growth under assumption of XoxF-MeDH driven conversion of formaldehyde to formate.

Network	O_2_ consumption rate (mmol g CDW^-1^h^-1^)	O_2_:CH_4_ consumption rates
Wild Type^∗^	13.77	1.18
Ratio between conversion of methanol into formaldehyde (CH_3_O) and formate (CHOOH):		
0.0 to CH_3_O / 1.0 to CHOOH	19.14	1.64
0.25 to CH_3_O / 0.75 CHOOH	17.39	1.49
0.5 to CH_3_O / 0.5 to CHOOH	16.17	1.38
0.75 to CH_3_O / 0.25 to CHOOH	14.95	1.28


*^∗^Flux balance analyses were carried out using modified computational model of methane metabolism (Akberdin et al., 2018). Methane uptake is set to 11.7 mmol g CDW^-*1*^h^-*1*^.*

## Discussion

The growth and activity of methylotrophic bacteria possessing only XoxF-MeDHs strictly depend on REEs (Keltjens et al., 2014; Pol et al., 2014). Methanotrophic bacteria which possess both xoxF-MeDH and mxaFI-MeDH systems tightly control expression of *mxaFI*, and switch to the xoxF enzyme when REEs are available (Chu et al., 2016; Yu et al., 2017; Huang et al., 2018; Zheng et al., 2018). Here we show that the La-supplementation affects the growth and methane consumption rates in *M.alcaliphilum* 20Z^R^. The physiological parameter changes suggest modification of the global metabolic networks beyond a simple substitution of the one PQQ-dependent enzyme with another. To uncover the high growth rate paradox in La-supplemented cells we compiled a set of *omic*-studies, including gene expression, proteomics and metabolomics.

The whole-genome transcriptomic data did not show any significant alterations in central metabolic pathways except the switch of the primary methanol oxidation system. It should be mentioned, that while MxaFI-MeDH, and associated cytochrome c_L_ are tremendously downregulated at both the transcript and proteins levels when 20Z^R^ cells are grown with La, XoxF-MeDH is upregulated only twofold. This implies that XoxF-MeDH might be involved in methane assimilation even under Ca-growth conditions. Taking into account the total MeDH protein counts, less XoxF-enzyme is needed to completely substitute for the MxaFI-MeDH function during La-growth. Together with increased rates of methane consumption, these observations suggest that the XoxF enzyme is more efficient than MxaFI. However, it could be speculated that the XoxF system requires higher input of methane, since the growth rate of La-supplemented cells reduced upon methane scarcity. The data indicate that XoxF operates differently than MxaFI *in vivo*.

The XoxF enzyme from 20Z^R^ is a typical XoxF5 enzyme usually found in Gammaproteobacteria. It has been proposed that the electrons from XoxF5 are transferred to a putative cytochrome cbb3-type (xoxG4, Yu et al., 2017). In this study we do not observe any correlation between XoxF and cytochrome cbb3 expression, indicating that the protein might have a different function in *M. alcaliphilum* 20Z^R^. Among all electron transfer systems, four cytochromes showed some response to La, but only one of them, cytochrome P460, was detected at the protein level. This cytochrome’s activity has been associated with the second step of ammonia oxidation (Bergmann and Hooper, 1994; Cua and Stein, 2011; Caranto et al., 2016); however, its function in methanotrophic bacteria remains elusive (Zahn et al., 1994; Bergmann et al., 1998). Similarly to XoxF, the enzyme is constitutively expressed in various methanotrophs, and it might represent an alternative electron acceptor for the enzyme. To confirm this, the function must be validated via mutagenesis. Nevertheless, the observed abundance of XoxG4 or P460 could not enable the same tight coupling observed for MxaFI and MxaG. One could speculate that XoxF transfers electrons to yet unknown system and/or to pMMO via direct electron coupling or reverse electron transfer. Taking into the account that the total number of XoxF peptides never reaches the same level as MxaFI, yet methane consumption rates increase, it is possible that the direct coupling between XoxF and pMMO is more efficient than the coupling between MxaFI and pMMO. Activation of the fatty acid degradation pathways upon growth with La, as a proxy for reduction of needs for intracytoplasmic membranes for MeDH:pMMO coupling (Culpepper and Rosenzweig, 2014), provide additional support for this idea. On the other hand, La-grown cells showed higher abundances of complex III (cytochrome bc_1_) proteins, which also opens up a possibility of more efficient reverse transfer. Overall, the abundances of cytochromes dropped slightly from 80 in Ca-grown cells to 77 in La-grown cells. Beside *mxaG* (detected only in Ca-grown cells) and *xoxG4*, two cytochromes, c6 and b557.5 were prevalent at the transcript levels in both Ca and La grown cells. The gene expression levels of the cytochromes C6 and b557.5 were contrary to each other, with cytochrome c6 being more prevalent upon methane-limiting growth (479.5 FPKM at optimal vs. 1507.7 FPKM at methane-limiting conditions), while b557.5 was highly expressed at optimal CH_4_:O_2_ supply (1785.6 FPKM at optimal vs. 192.2 FPKM at methane-limiting conditions). Cytochrome b557.5 might represent an equivalent of cytc_H_, which links MeDH-associated cytochromes to complex IV (Anthony and Williams, 2003). However, it should be noted, that no peptides matching *b*557.5 were detected. Cytochrome c6 was also the most prevalent electron carrier in proteome. The cytochrome is known as a redox carrier in phototrophic organisms, which transfers electrons from cyt*bf* to photosystem I (Gupta et al., 2002). In this study, the expression of the cytochrome c6 could be connected with reduced methane supply and/or oxygenation level. However, the cytochrome was the most abundant cytochrome at protein level at all growth conditions, which makes the cytochrome the best candidate for transferring electrons to pMMO from bc1 when direct coupling is not possible (Akberdin et al., 2018). This role of the cytc6 is being validated via mutagenesis.

The gene expression profiles complemented by protein-abundance and metabolomics data highlight a set of possible post-transcriptional alterations in metabolic networks. The higher abundance of TCA/serine cycle enzymes and intermediates might be linked to increased carbon flow through those pathways. The data are consistent with the physiological data indicating that La-cells consume more methane carbon and produce more CO_2_ per unit of biomass. Taken together, these data suggest that the substitution of Ca with La impacts the amount of NADH available for biosynthesis and/or the amount of carbon accessible for assimilation. One plausible explanation for these changes is a possible direct conversion of formaldehyde to formate by XoxF-MeDH. Both metabolomics and the flux-balance simulations further strengthen this hypothesis (Figure 1). The metabolomics profiles of La-grown cells could be best modeled by an assumption that 75% of the methanol is converted to formaldehyde, while 25% is converted into formate (the Spearman’s index of 0.6, *p*-value = 8E–06).

La-growth is strongly associated with overexpression of two additional systems: a putative sulfate transporter (>70-fold increase) and Fae2, a formaldehyde activating enzyme (14-fold increase). A strong correlation between La-supplementation and the transporter expression suggests that the system might contribute to REE rather than sulfate acquisition. Several activities have been previously hypothesized for Fae-homologs, ranging from methyl-group sensing to reverse conversion of methylene-tetrahydrofolate back to formaldehyde for incorporation into the RuMP pathway (Good et al., 2015). Taking into account the possibility of increased flux into formate in La-grown cells and the increase in the abundance of H_4_folate pathway enzymes, the latter might justify the activation of an alternative Fae in *M. alcaliphilum* 20Z^R^.

Overall, our study provides a global overview of the Ca/La-switch on metabolic networks in *M. alcaliphilum* 20Z^R^ (summarized in Figure 1). We found that the XoxF-MeDH system provides a higher growth rate, while the MxaFI-MeDH system enables more efficient methane utilization in *M. alcaliphilum* 20Z^R^ and likely other gammaproteobacterial methanotrophs. The mechanism underlining the physiological outputs includes a number of alterations in metabolic networks, navigated by a redox swap. While La-grown cells receive a boost from more efficient coupling between pMMO and XoxF, as well as extra electron flow toward methane oxidation due to conversion of methanol to formate, they are limited in redox power. On another hand, Ca-grown cells are more balanced with respect to redox demand and their slow growth could be explained by less efficient coupling between pMMO and MxaFI. Together, these data suggest that cells possessing both enzymes would have advantages in highly dynamic and competitive environmental niches.

A number of novel proteins as well as new metabolic connections for enzymatic systems with elusive functions in methanotrophy were uncovered. The validation of the predictions arising from these global analyses awaits further investigation of factors contributing to the changes, including the identification of XoxF-MeDH electron transfer partner (including XoxF-pMMO coupling), the description of the putative La-induced transporters and the enzymatic characterization of Fae2, cytochromes bc1, P460, and c6 functions.

## Materials and Methods

### Strain and Growth Media

*M. alcaliphilum* 20Z^R^ cells were grown using P media (g/L) (Akberdin et al., 2018): KNO_3_, 1; MgSO_4_ × 7H_2_O, 0.2; NaCl, 30; CaCl_2_ × 2H_2_O, 0.02; or LaCl_3_ × 7 H_2_O, 0.07; and supplemented with 1 ml/L of trace element solution, 20 ml/L of phosphate solution (5.44 g KH_2_PO_4_; 5.68 g Na_2_HPO_4_) and 40 ml/L of 1 M carbonate buffer.

### Cultivation and Bioreactor Parameters

Culturing was carried out in either closed vials (batch cultures) or bioreactor cultures (fed-batch or continuous culture). Batch cultures were grown in 125 ml, 250 ml, or 1.2 L bottles with shaking at 200 r.p.m. The headspace:medium ratio was set at 4:1. Methane (99.9%, Airgas) was injected into vials to represent 20% of the headspace. Samples of batch cultures were used for metabolomics studies.

A DASbox mini bioreactor (0.5 L working volume; 250 ml culture) with two individual bioreactor units, each having automatic temperature, pH, and DO controls, a sample port for measuring OD, and a coupling to a BlueSens sensor system for simultaneous measuring off-gasses (CH_4_, O_2_, and CO_2_) were used for bioreactor cultures. The bioreactor set-up is shown in Supplementary Figure S1. The following pre-mixed gas mixtures were used for bioreactor studies: (i) 5% CH_4_ :5 % O_2_, to represent optimal growth; and (ii) 2.5% CH_4_ : 10% O_2_ to represent methane-limiting conditions. Gas tanks were connected to a mass flow controller and the gas mixture was directly purged into the bioreactor culture at 0.2–1 sL h^-1^ rates. In batch cultures, methane (99.9%, Airgas) was injected into vials to represent 20% of the headspace. The methane and oxygen consumption and CO_2_ production rates were calculated by estimating the decline (or increase) of the corresponding compounds over time. The data were analyzed to assess yield (Y), growth rate, and O_2_/substrate ratios. Samples of bioreactor cultures were collected for metabolomic, proteomics and transcriptomic studies.

### RNA Sequencing and Analysis

Samples (45 ml) of bioreactor cultures, La-optimum, La-CH4 limited, Ca-optimum and Ca-CH4 limited, were collected and immediately transferred into tubes containing 5 ml of the stop solution (5% water-equilibrated phenol in ethanol) (Griffiths et al., 2000). Cells were pelleted by centrifugation at 4700 rpm for 15 min, and RNA was extracted using a RNeasy kit and treated with PureLink DNaseI (ThermoFisher Scientific) according to the manufacturer’s instructions. Samples were sequenced on an Illumina HiSeq2500 with ∼50 million/sample SR50 reads by IGM Genomics Center, University of California, San Diego. All experiments were performed with at least two biological replicates.

The quality of the obtained raw Fastq files was checked and analyzed with FastQC^1^. To improve the quality of the raw reads we employed the Trimmomatic tool (Bolger et al., 2014) using these procedures: removing a base from either the start or end position if the quality was low; trimming bases on a sliding window method; removing any remaining reads that are <36 bases long. The trimmed reads were aligned to the annotated *M. alcaliphilum* 20Z^R^ genome as retrieved from the NCBI database (the latest genome build ASM96853v1) on January 18, 2018 (Vuilleumier et al., 2012). Alignment was performed using TopHat2 (Kim et al., 2013). The alignments were post-processed into sorted BAM files with SAMTools version 1.4 (Li et al., 2009). Reads were attributed to open reading frames (ORFs) using the htseq-count tool from the “HTSeq” framework version 0.7.2 (Anders et al., 2015) based on gtf files with coordinates of genes from ASM96853v1 and indexed SAM file. Differential expression analysis was performed with DESeq2 1.16.1 (Love et al., 2014) using R 3.4.1. Principal component analysis of the normalized logarithmic transformed read counts was used by means of DESeq2 (Anders and Huber, 2010) in order to determine the reproducibility of analyzed replicates (Supplementary Figure S2). Genes were considered to be differentially expressed if they had an average change of greater than 1.5-fold when comparing normalized counts as well as an adjusted *p*-value of less than 0.05 to ensure statistical significance (Anders and Huber, 2010). We also applied an alternative Rockhopper 2 tool with default parameters to confirm the robustness of the results (Tjaden, 2015).

### Proteomics Study

Biomass was harvested by centrifuging 50 ml of culture for each technical replicate at 4000 rpm for 20 min. Cells pellets were frozen and stored at -80°C. SDS-lysis buffer [4% Sodium dodecyl sulfate (SDS) (w/v), 100 mM Tris–HCl pH 7.6, 100 mM dithiothreitol (DTT)] was added to the pellets, vortexed into solution and fractions (100 μl) transferred to 1.5 mL centrifuge tubes. Each sample was incubated at 95°C for 5 min to completely lyse the cells and reduce and denature the protein. The samples were cooled at 4°C for 30 min and centrifuged at 15,000 × *g* for 10 min to pellet any remaining debris. Filter Aided Sample Preparation (FASP) (Wiśniewski et al., 2009) kits were used for protein digestion (Expedeon, San Diego, CA, United States) according to the manufacturer’s instructions. Briefly, 400 μl of 8 M urea (all reagents included in the kit) was added to each 500 μl 30 K molecular weight cut off (MWCO) FASP spin column and 50 μl of the sample in SDS buffer was added, centrifuged at 14,000 × *g* for 30 min to bring the sample all the way to the dead volume. The waste was removed from the bottom of the tube and another 400 μl of 8 M urea was added to the column and centrifuged again at 14,000 × *g* for 30 min and repeated once more. 400 μl of 50 mM ammonium bicarbonate (ABC) was added to each column and centrifuged for 30 min, repeated twice. The column was placed into a new fresh, clean and labeled collection tube. Digestion solution was made by dissolving 4 μg trypsin in 75 μL 50 mM ABC solution and added to the sample. Each sample was incubated for 3 h at 37°C with 800 rpm shaking on a thermomixer with a thermotop (Eppendorf, Hamburg, Germany) to reduce condensation into the cap. The resultant peptides were then centrifuged through the filter and into the collection tube at 14,000 × *g* for 15 min. The peptides in the collection tube were snap frozen in liquid N_2_ and the column placed back into a new collection tube and digested again overnight with 150 μL of digestion solution. The following day the peptides were spun out and added to the 3 h peptide collection tube, the samples were then concentrated to ∼30 μL using a SpeedVac. Final peptide concentrations were determined using a bicinchoninic acid (BCA) assay (Thermo Scientific, Waltham, MA, United States). All of the samples were diluted to 0.2 μg/μl for MS analysis.

Peptides were resuspended in water and a total of 500 ng were analyzed by liquid chromatography-tandem mass spectrometry (LC-MS/MS) on Waters nano-Acquity M-Class dual pumping UPLC system (Milford, MA, United States) connected to a Q-Exactive HF mass spectrometer (Thermo Scientific, San Jose, CA, United States) as described in detail elsewhere (Yang et al., 2017). LC-MS/MS data was processed with DeconMSn (Mayampurath et al., 2008) and peptide identification was performed using MS-GF+ (Kim and Pevzner, 2014) using the following parameters: (1) tryptic digestion in a least one terminus of the peptide, (2) 20 ppm parent ion mass error tolerance, and (3) methionine oxidation and lysine trimethylation as variable modifications. Identifications were filtered with a probability score ≤x1e-9, resulting on a false-discovery rate ≤1% at the protein level. The number of spectra that mapped to each protein were counted that total is then reported as spectral count. The number of observed spectra were then determined using a proxy of relative abundance of proteins. The number of spectra observed were averaged across replicates and a fold-change of greater than 2 was considered significant.

### Non-Targeted Metabolite Profiling

Metabolomic analyses of cells and spent supernatant from cultures of the *M. alcaliphilum* 20Z^R^ grown on Ca or La were performed according to the published protocol (Akberdin et al., 2018).

### Flux Balance Analysis With COBRA

A recently published genome-scale model of *M. alcaliphilum* 20Z^R^ (Akberdin et al., 2018) was used to simulate the Ca-REE switch. To consider the functional activity of XoxF-MeDH, a reaction (Reaction ID: “MXALa”) representing cytochrome-mediated conversion of formaldehyde into formate was included. The updated model is available on the web-site: http://sci.sdsu.edu/kalyuzhlab/.

### Urea Analysis

Cultures of *M. alcaliphilum* 20*Z^R^* were grown with Ca or La (3 biological replicates per experiment) and methane as a carbon source, in closed vials (25 ml) to an OD 1 to reproduce bioreactor settings. Cells were transferred into tubes to pellet the cells by centrifugation at 4700 rpm for 15 min. The supernatant was collected and then tested for urea using a Urea kit (QuantiChrom Urea assay kit DIUR-100) following the manufacturer’s procedure. The reactions were measured using a 96-well plate reader spectrophotometer synergy HT (Biotek) with two technical replicates for each specific environment. The results were compared to a standard created using the kits procedure.

## Author Contributions

MK designed and coordinated the study. IA and MK analyzed the data and wrote the first draft of the manuscript. RH and DC performed cultivation experiments, and prepared samples for proteomics, RNAseq and metabolomics. DO and IA conducted RNA-seq analysis. CN, AS, EN, and JA carried out proteomics study. All authors read and approved the final manuscript.

## Conflict of Interest Statement

The authors declare that the research was conducted in the absence of any commercial or financial relationships that could be construed as a potential conflict of interest.

## Footnotes

1 http://www.bioinformatics.babraham.ac.uk/projects/fastqc/

## Supplementary Material

FIGURE S1 Schematic components of a continuous culture bioreactor. MTU-Mass Transfer Unit; MSP-Manual Sample Port; MFM-Mass Flow Meter;BS-Bluesens sensors. The protocol is adapted for working with a mini-parallel bioreactor system, such as DasBox (Eppendorf). The system is connected to a custom-built gas-distribution system, which controls the gas-mixture input. Only non-flammable mixtures of methane (5% or 2.5% CH_4_) and oxygen (2.5–5% O_2_) were used, and output gasses must be connected to exhaust vents. The DasBox system offers single use plastic vessels, which are handy for small-scale analyses of minerals (Cu, Fe, and La, etc.,) effects on cell growth and/or methane oxidation. Four parallel experiments have been carried in one run, providing sufficient statistical data for analysis.

FIGURE S2 **(A)** Principal component analysis of analyzed transcriptomic datasets. Individual samples are indicated according to the next notation: light red circle – Ca-regulated growth, blue circle – La-regulated growth; **(B)** The MA-plot shows the log2 fold changes between Ca- and La-regulated growths of 20 Z over the mean of normalized counts. The *x*-axis represents the average expression of genes over samples and the *y*-axis represents the log2 fold change between the Ca-CH4 and La-CH4 growth conditions. Red circles represent differentially expressed genes with statistical significance, *p* < 0.05.

TABLE S1 Complete list of differentially expressed genes.

TABLE S2 Proteomics datasets.

TABLE S3 Metabolomics dataset.
