# Supplementary figures and images for: Rare Earth Elements Alter Redox Balance in Methylomicrobium alcaliphilum 20ZR
Source: Front Microbiol. 2018 Nov 27;9:2735. doi: 10.3389/fmicb.2018.02735 (PMC6277846; doi:10.3389/fmicb.2018.02735)

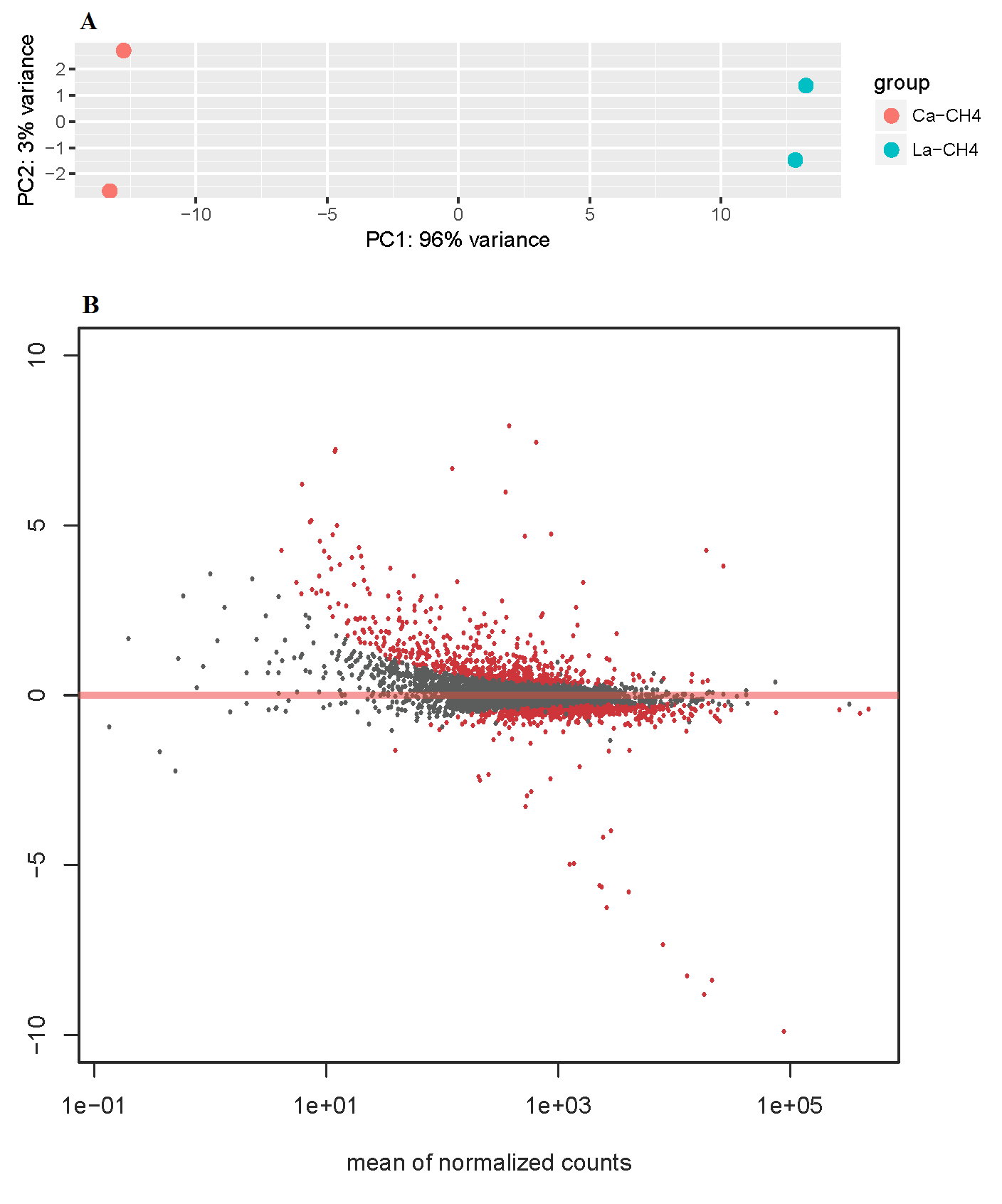

Supplement: FIGURE S1 — Schematic components of a continuous culture bioreactor. MTU-Mass Transfer Unit; MSP-Manual Sample Port; MFM-Mass Flow Meter;BS-Bluesens sensors. The protocol is adapted for working with a mini-parallel bioreactor system, such as DasBox (Eppendorf). The system is connected to a custom-built gas-distribution system, which controls the gas-mixture input. Only non-flammable mixtures of methane (5% or 2.5% CH4) and oxygen (2.5–5% O2) were used, and output gasses must be connected to exhaust vents. The DasBox system offers single use plastic vessels, which are handy for small-scale analyses of minerals (Cu, Fe, and La, etc.,) effects on cell growth and/or methane oxidation. Four parallel experiments have been carried in one run, providing sufficient statistical data for analysis. [file Image_1.PNG]

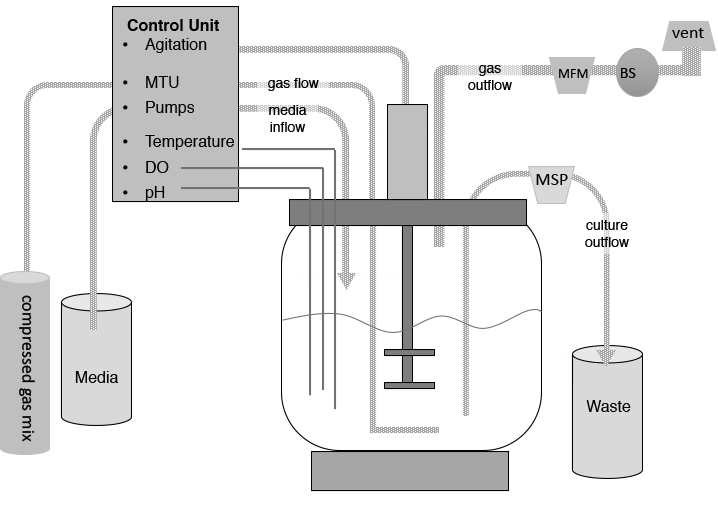

Supplement: FIGURE S2 — (A) Principal component analysis of analyzed transcriptomic datasets. Individual samples are indicated according to the next notation: light red circle – Ca-regulated growth, blue circle – La-regulated growth; (B) The MA-plot shows the log2 fold changes between Ca- and La-regulated growths of 20 Z over the mean of normalized counts. The x-axis represents the average expression of genes over samples and the y-axis represents the log2 fold change between the Ca-CH4 and La-CH4 growth conditions. Red circles represent differentially expressed genes with statistical significance, p < 0.05. [file Image_2.TIF]
